# Canis MitoSNP database: a functional tool useful for comparative analyses of human and canine mitochondrial genomes

**DOI:** 10.1007/s13353-023-00764-w

**Published:** 2023-06-23

**Authors:** Krzysztof Kowal, Angelika Tkaczyk-Wlizło, Marcin Jusiak, Ludmiła Grzybowska-Szatkowska, Brygida Ślaska

**Affiliations:** 1 Institute of Biological Bases of Animal Production, University of Life Sciences in Lublin, Akademicka 13 St., 20-950 Lublin, Poland; 2 Department of Radiotherapy, Medical University of Lublin, Chodźki 7, 20-093 Lublin, Poland

**Keywords:** mtDNA, Bioinformatics, Database, SNP, Dog, Human

## Abstract

*Canis* MitoSNP is a tool allowing assignment of each mitochondrial genomic position a corresponding position in the mitochondrial gene and in the structure of tRNA, rRNA, and protein. The main aim of this bioinformatic tool was to use data from other bioinformatic tools (TMHMM, SOPMA, tRNA-SCAN, RNAfold, ConSurf) for dog and human mitochondrial genes in order to shorten the time necessary for the analysis of the whole genome single nucleotide polymorphism (SNP) as well as amino acid and protein analyses. Each position in the canine mitochondrial genome is assigned a position in genes, in codons, an amino acid position in proteins, or a position in tRNA or rRNA molecules. Therefore, a user analysing changes in the canine and human mitochondrial genome does not need to extract the sequences of individual genes from the mitochondrial genome for analysis and there is no need to rewrite them into amino acid sequences to assess whether the change is synonymous or nonsynonymous. *Canis* mitoSNP allows the comparison between the human and canine mitochondrial genomes as well. The Clustal W alignment of the dog and human mitochondrial DNA reference sequences for each gene obtained from GenBank (NC_002008.4 dog, NC_012920.1 human) was performed in order to determine which position in the canine mitochondrial genome corresponds to the position in the human mitochondrial genome. This function may be useful for the comparative analyses. The tool is available at: https://canismitosnp.pl.

**Supplementary Information:**

The online version contains supplementary material available at 10.1007/s13353-023-00764-w.

## Introduction

The recent decade has witnessed an explosion in the amount of available biological sequence data due to the rapid progress of high-throughput sequencing projects. However, the amount of biological data is becoming so vast that traditional data analysis platforms and methods can no longer meet the need to rapidly perform data analysis tasks in life sciences (Yin et al. 2017). With this exponential growth of sequence data, rich biological data analytics applications, such as sequence alignment (including short read alignment), genome assembly, single nucleotide polymorphism (SNP) detection, and genome-wide association study (GWAS) are developed and studied. Such applications as SNP detection (Li et al. 2009) and GWAS (Nielsen et al. 2012) may also take days or even months to finish processing one dataset. Although these analyses may not be as time-consuming for mitochondrial genomes as for nuclear genomes, there is a necessity of fast and user-friendly bioinformatic tools facilitating complex genomic analyses and shortening the time necessary to obtain the results.

Dogs live close to humans or even in the human environment. They are exposed to similar risk factors, thus the aetiology and pathogenesis of several diseases is likely to be similar to that of human diseases (Khanna et al. 2006; Pinho et al. 2012; Ślaska et al. 2013; Switonski et al. 2004). Dogs naturally develop the same mitochondrial diseases as humans, e.g. cancers, mitochondrial myopathies, or encephalopathies; therefore, they are appropriate model animals to analyse molecular changes observed during human disease development (Tkaczyk-Wlizło et al. 2022).

There are few databases specifically dedicated to dogs. For the hypervariable region I (HVI) haplotypes, the database *Canis* mtDNA HVI was created (http://chd.vnbiology.com/) (Thai et al. 2017). The mtDNA haplogroups of *Canis lupus familiaris* were described in http://clf.mtdna.tree.cm.umk.pl/ (Duleba et al. 2015), whereas the database DoGSD (http://dogsd.big.ac.cn) focuses on whole genome SNP data from domesticated dogs and grey wolves. In Ensembl (http://www.ensembl.org/Canis_lupus_familiaris/Info/Annotation) and NCBI Entrez databases (http://www.ncbi.nlm.nih.gov/projects/mapview/static/dogsearch.html), dog genomes are available including genetic information on mitochondrial genomes.

As dogs are genetic model species, e.g., for cancer diseases (Kowal et al. 2021; Tkaczyk-Wlizło et al. 2022), there are databases with specific SNP-STR/microsatellite compound markers in canine along with human STRs (http://www.sbg.bio.ic.ac.uk/~ino/SNPSTRdatabase.html) (Agrafioti & Stumpf 2007). Furthermore, the Immuno Polymorphism Database (http://www.ebi.ac.uk/ipd/) is included in a set of specialist databases related to the study of polymorphic genes in the immune system of several animals, including dogs (Robinson et al. 2013). For proteins, there is a database collecting information on protein signatures of *Canis lupus familiaris* diseases called CanisOne (Fernandes et al. 2016). The human mitochondrial genome is described on the https://www.mitomap.org/MITOMAP database website (Lott et al. 2013). However, to date, there has been no database focused solely on the canine mitochondrial genome and its similarities to the human mtDNA genome.

The main aim of this bioinformatic tool was to use data from other bioinformatic tools, including TMHMM (TransMembrane prediction using hidden Markov Models (Hallgren et al. 2022)), SOPMA (SOPMA secondary structure prediction method (Combet et al. 2000)), trnan scan (Lowe & Chan 2016), RNAfold (Gruber et al. 2008), and ConSurf (Ashkenazy et al. 2016) for dog and human mitochondrial genes in order to shorten the time necessary for the analysis of the whole genome single nucleotide polymorphism as well as primary and secondary protein structure analyses. Moreover, the tool facilitates the comparison between human and canine mitochondrial genome reference sequences in order to find similarities and differences that can facilitate further comparisons of the results obtained by the users. The tool is available on the https://canismitosnp.pl/ website.

## Database construction

### Data source

In the Canis MitoSNP tool, the information from five bioinformatic tools was used. The information about genomic positions for each gene, genomic sequences for both strands, gene length, amino acid length, and amino acid positions in protein was downloaded from the GenBank database. Based on this data, the codons, and positions of nucleotides in the codons were determined. For tRNA molecules, the secondary structure was predicted using the tRNAscan tool (Lowe and Chan 2016) and confirmed with the annotation proposed by Pütz et al. (2007). The secondary structure of 12S and 16S rRNA molecules was predicted with the use of the RNAfold tool (Gruber et al. 2008) by evaluating minimum free energy prediction (FEP) at 37 °C and by evaluating thermodynamic ensemble prediction (TEP) at the same temperature. The features of proteins were determined using SOPMA for secondary structure prediction (Combet et al. 2000), Deep TMHMM for transmembrane domain structure prediction (Hallgren et al. 2022), and ConSurf for evaluation of functional and structural regions, buried or exposed residues, and assessment of the conservation grade (Ashkenazy et al. 2010, 2016). The reference sequences of human and dog mtDNA genes and proteins were used in each tool. The collected data was organised in the Excel database along with the information obtained from GenBank.

### Alignment and comparison of human and dog mtDNA genes

The reference sequences of human and dog mitochondrial genomes were obtained from GenBank (NC_012920.1 and NC_002008.4, RefSeq assembly accession: GCF_000002285.5). Each of the 37 human and dog mtDNA genes were separately aligned and compared with the use of the Unipro uGene tool (v.37.0) (Okonechnikov et al. 2012) CLUSTAL W algorithm (gap opening penalty = 15.00; gap extension penalty = 6.66; weight matrix = IUB; iteration type = NONE; max iterations = 3). Especially in the case of the 12S rRNA and 16S rRNA molecules, alignments with the highest homology rate were chosen. Protein-coding genes were translated to amino acid sequences and compared with protein reference sequences. The alignment of each human and dog amino acid sequence was performed with the use of the CLUSTAL W algorithm as well (gap opening penalty = 10.00; gap extension penalty = 0.20; weight matrix = BLOSUM; iteration type = NONE; max iterations = 3; gap separation distance = 4).

### Alignment and comparison of the non-coding regions of the human and dog mtDNA genomes

The information on the localisation of human non-coding regions, i.e., MT-7SDNA, MT-HV1, MT-HV2, MT-HV3, MT-OHR, MT-CSB1, MT-CSB2, MT-CSB3, MT-TFX, MT-TFY, MT-4H, MT-3H, MT-LSP, MT-TFL, MT-TFH, MT-TAS1, MT-TAS2, MT-5, and MT-3L, was obtained from the https://www.mitomap.org/MITOMAP database (Lott et al. 2013). The alignment of the abovementioned non-coding sequences with the canine D-loop sequence or the mtDNA genome was done with the use of the CLUSTAL W algorithm (gap opening penalty = 15.00; gap extension penalty = 6.66; weight matrix = IUB; iteration type = NONE; max iterations = 3;). Although there was no information about the location of these non-coding regions in the canine reference mtDNA genome, we compared the sequences of these two organisms and indicated the localisations of homological positions in the dog genome taking into account the H and L strand and the placement of other genes and regions in the human mtDNA genome (Supplementary Table). Browsing the human genome, the user can find the exact positions for non-coding regions, but these regions were not indicated in the canine mtDNA genome, as they were not determined experimentally.

### Annotation of human and dog positions in mRNA, tRNA, and rRNA genes

Each gene position in the human mtDNA sequence was determined according to the numbering in the revised Cambridge Reference Sequence (rCRS, NC_012920.1), and the numbering in the canine mtDNA genes was determined according to the positions in the reference sequence. In the case of tRNA genes, the numbering and positions of structural domains were determined according to the Mamit-tRNA database (Pütz et al. 2007). If there was a gap in the human or dog nucleotide sequence or the amino acid sequence, this position was omitted in the numbering. Each genomic position which corresponded to two genes or regions, i.e., the *ND4/ND4L* region, was marked with an asterisk (*) and described separately for each gene. All the positions in the database have their own ID number, which is non-informative for users as it is only for the purpose of record ordering in the database.

### Canis SNP finder content

*Canis* MitoSNP, the canine mitochondrial DNA database, is composed of four separate pages: *Canis* SNP finder, tRNA properties, mRNA properties, and protein properties. The main functionality of the website is to facilitate finding the information about the exact position(s) in the whole genome and/or specific genes of human and canine mtDNA. The user can find chosen positions in either the human or canine mitochondrial genome (Supplementary Fig. 1a).

Additionally, the browser demonstrates whether the position/s is/are identical or different in the other genome. If the user wishes to find information about some positions in a specific gene, there is such a possibility in point 2. After choosing option 2 “SNP position in the specific gene,” the user has a possibility to choose a gene of interest (Supplementary Fig. 1a). Depending on the organism selected in point 1, the user will be presented a list of genes and regions in the genome. In point 3, the user may choose either one position or several positions separated by commas. In the case of selection of consecutive positions, the user should fill the field with the first and the last position of the range separated by the hyphen (Supplementary Fig.  1a). If the user wishes to see all the results for the chosen gene, the option “Show all” must be selected.

After clicking the “Search” button, the user will be presented the Results table (Supplementary Fig. 1b). The number of columns in the Results table depends on the region/gene where the position is localised. For all (tRNA-coding, rRNA-coding, and protein-coding) positions, the following columns are presented: ID (non-informative for the user) genome position, dog mtDNA 5′–3′ strand, dog mtDNA 3′–5′ strand, type/region, gene, gene position, human mtDNA position, human mtDNA ref. seq., identical/different, and human gene/region (Supplementary Fig. 1b). The tRNA-SCAN column is shown for tRNA-coding positions, whereas the secondary structure of FEP at 37°and the secondary structure of TEP at 37°columns are presented for rRNA-coding positions. The following columns: codon, position in codon or region, amino acid (aa) position in protein, amino acid 1-letter, amino acid 3-letter, SOPMA, TMHMM, conservation grade, buried or exposed residue, and functional or structural residue are shown for protein-coding positions. Therefore, the user is able to obtain complete information about the localisation of a position in the genome, in a specific gene, and in the secondary structure of a protein at the same time. In addition, the tool presents data for both genes if the position is part of two separate genes. The “Results” table may be easily downloaded as an.xlsx file on the user’s computer upon clicking on “download xlsx file.”

### tRNA properties

The “tRNA properties” webpage is useful for users analysing changes in the secondary structure of human and canine mitochondrial tRNA genes. There are 22 tRNA genes encoded in mammalian mitochondrial genomes (Kim et al. 1998), from which eight are encoded on the complement (heavy) strand. The users are informed about the positions of tRNA-coding genes in both genomes as well as the length of these genes. The analysis of the homology between the tRNA genes of these two organisms revealed how many transitions, transversions, and gaps differentiate these genes. The user may compare the percentage of homology between human and canine mt-tRNA genes (Supplementary Fig. 2a). The highest homology rate was observed for the *MTTM* gene (97%), whereas the lowest score was observed for *ex aequo* the *MTTT* and *MTTQ* genes (65%).

Upon clicking on the highlighted gene name of interest, the user is able to see the detailed secondary structure of the canine and human tRNA gene as well as the detailed description of each position of these two tRNA-coding genes (Supplementary Fig. 2b). The data in the table can be downloaded upon clicking on the “download xlxs” button.

### mRNA properties

As in the case of tRNA properties, the “mRNA properties” website allows the user to perform a comparative analysis of protein-coding genes, their positions on the genomes, their length, and the number of differences between the human and canine genomes. The highest homology rate observed for protein-coding genes was 77% (*MT-CO1*), whereas the lowest rate was observed for *ex aequo MT-ND6* and *MT-ND2* (64%). For each gene, detailed information on the identical and different positions in human and canine protein-coding genes are available upon clicking on the highlighted gene name (Supplementary Fig. 3a). The user is able to compare both genes encoded on the human and canine genomes and verify which amino acid is encoded by each position without the necessity of translating the sequence in another tool (Supplementary Fig. 3b).

### Protein properties

The “protein properties” website facilitates the comparison of amino acids in proteins encoded on the mitochondrial genome. The canine and human mtDNA genomes encode 13 protein-coding essential genes of the respiratory chain: seven subunits of complex I (*ND1*, *ND2, ND3*, *ND4*, *ND4L*, *ND5*, *ND6*), one subunit of complex III (*CYTB*), three subunits of cytochrome c oxidase (*COX1*, *COX2, COX3*), and two subunits of ATP synthase (*ATP6* and *ATP8*) (Tkaczyk-Wlizło et al. 2022). The amino acid sequence and composition may vary among these two described species; therefore, the user is informed about the identical positions in both proteins and the differences between them. The highest homology rate of amino acid sequences was observed in the case of the *MT-CO1* protein (92%), whereas the lowest homology was noted in the case of the *MT-ATP8* gene (53%) (Supplementary Fig. 4a). Upon clicking on “protein gene,” detailed information will be shown to the user (Supplementary Fig. 4b). The amino acids were classified according to Dagan et al. (2002). The classification by volume and polarity was made by dividing the amino acids into six categories: special (C), neutral and small (A, G, P, S, T), polar and relatively small (N, D, Q, E), polar and relatively large (R, H, K), nonpolar and relatively small (I, L, M, V), and nonpolar and relatively large (F, W, Y) (Dagan et al. 2002). Based on the properties, we indicated conservative and non-conservative differences between human and canine proteins. The user is able to download the whole table upon clicking on the download xlsx button.

## Discussion

The *Canis* MitoSNP database is the first tool that facilitates a complex comparison between the human and canine mitochondrial genomes in each position. Such functionality is advisable for researchers studying dogs as model organisms in the case of human diseases. Moreover, the availability of data obtained from several reliable bioinformatic resources widens the scope of analyses. The users are able to find out whether the polymorphisms or mutations in the human and/or canine genomes cause nonsynonymous changes in the protein structure and whether these changes can alter the protein molecular stability or functionality. This option shortens the time necessary for obtaining protein sequences and analysing these sequences in other bioinformatic tools. However, it should be emphasised that the *Canis* MitoSNP database does not replace any of the tools from which the data was obtained (TMHMM, SOPMA, tRNA-SCAN. RNAfold, ConSurf) but helps to find information about proteins, tRNA, or rRNA structures from these bioinformatic tools without the necessity of performing these analyses. Therefore, the user obtains the information on the reference sequences which may compare with his results. Such a comparison can be used for screening and selecting most significant data for the next step of more complex analyses.

The functional regions of human mitochondrial DNA were identified in the previous century, i.e., MT-3H and MT-4H in 1995 (Suzuki et al. 1995), MT-TFX and MT-TFY in 1987 (Fisher et al. 1987), and MT-LSP and MT-HSP in 1985 (Hixson and Clayton 1985). In the case of canine functional regions, Kim et al. (1998) identified the site of origin of light strand replication (MT-OLR). Based on the comparison with human mtDNA as well as according to the plausible localisation on the light or heavy strand, we identified homological positions of the following functional regions in canine mtDNA: MT-3H, MT-3L, MT-4H, MT-5, MT-Hum, MT-TAS2, and MT-TER (Supplementary Table). Nevertheless, it should be emphasised that these functional regions should be confirmed experimentally as the sequence homology with human regions and the placement on the mitochondrial DNA may be not sufficient for proper identification of these regions. On the other hand, in our opinion, the information about the localisation of the homological positions in canine mtDNA to these human functional regions may be useful for better understanding of plausible functional regions that may be affected and elucidation of the functionality of the canine mitochondrial genome.

## Conclusions

Canis MitoSNP is the first known database gathering detailed information on the localisation of every single position in the canine mitogenome and assigning the corresponding position in the human mitogenome. The tool shortens the time necessary for the analysis of the secondary structure of mRNA, tRNA, and rRNA genes. The database is suitable for screening changes observed in the analysis of variants of human and dog mtDNA. Moreover, the tool is suitable for the analysis of changes observed in the primary and secondary structure of proteins.

## Supplementary Information

Below is the link to the electronic supplementary material.

Supplementary file1 (DOCX 3549 KB)

Supplementary file2 (DOCX 19 KB)

## Data Availability

All data is available on the website www.canismitosnp.pl.
