# Supplementary material for: Canis MitoSNP database: a functional tool useful for comparative analyses of human and canine mitochondrial genomes
Source: J Appl Genet. 2023 Jun 23;64(3):515–20. doi: 10.1007/s13353-023-00764-w (PMC10457218; doi:10.1007/s13353-023-00764-w)
Supplement: Supplementary file 1 — Supplementary file1 (DOCX 3549 KB) [file 13353_2023_764_MOESM1_ESM.docx]

**(1a)**


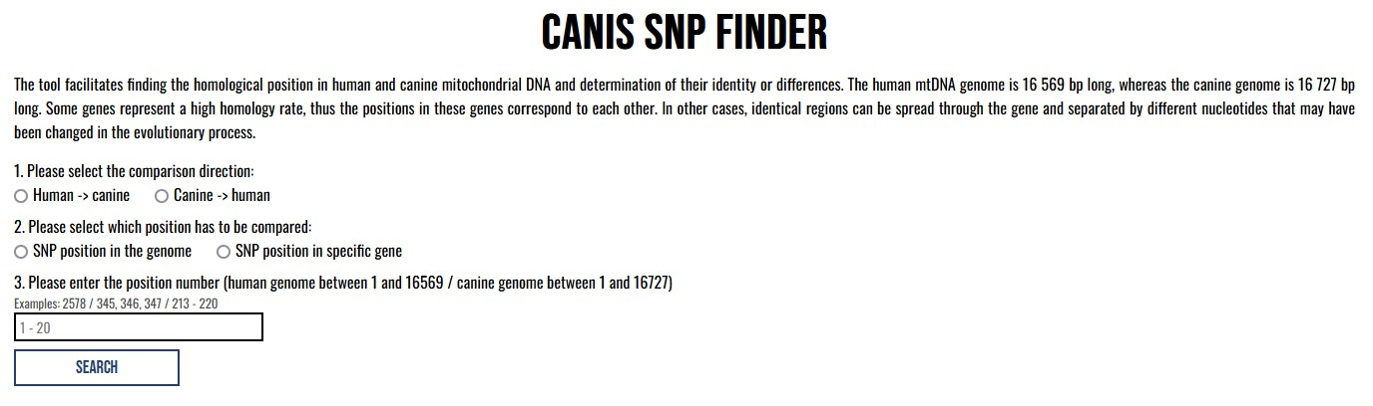


**(1b)**


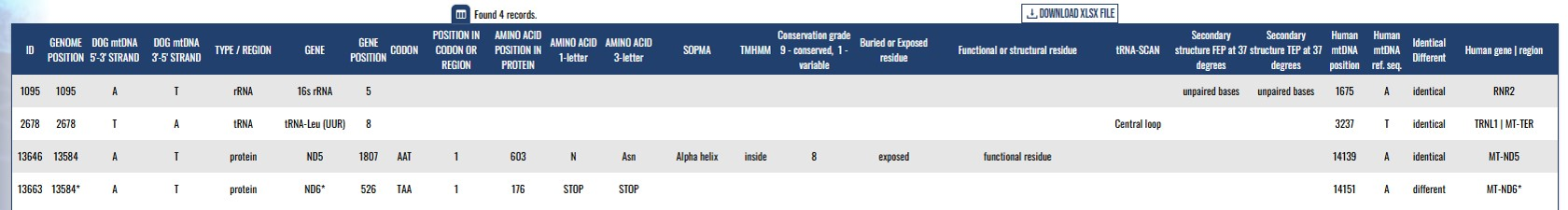


**Supplementary figures 1a.** Presentation of data after choosing the SNP position in a specific gene. **1b.** The Results window for three genomic (1095, 2678, 13584) positions in the canine mitochondrial genome.

**(2a)**


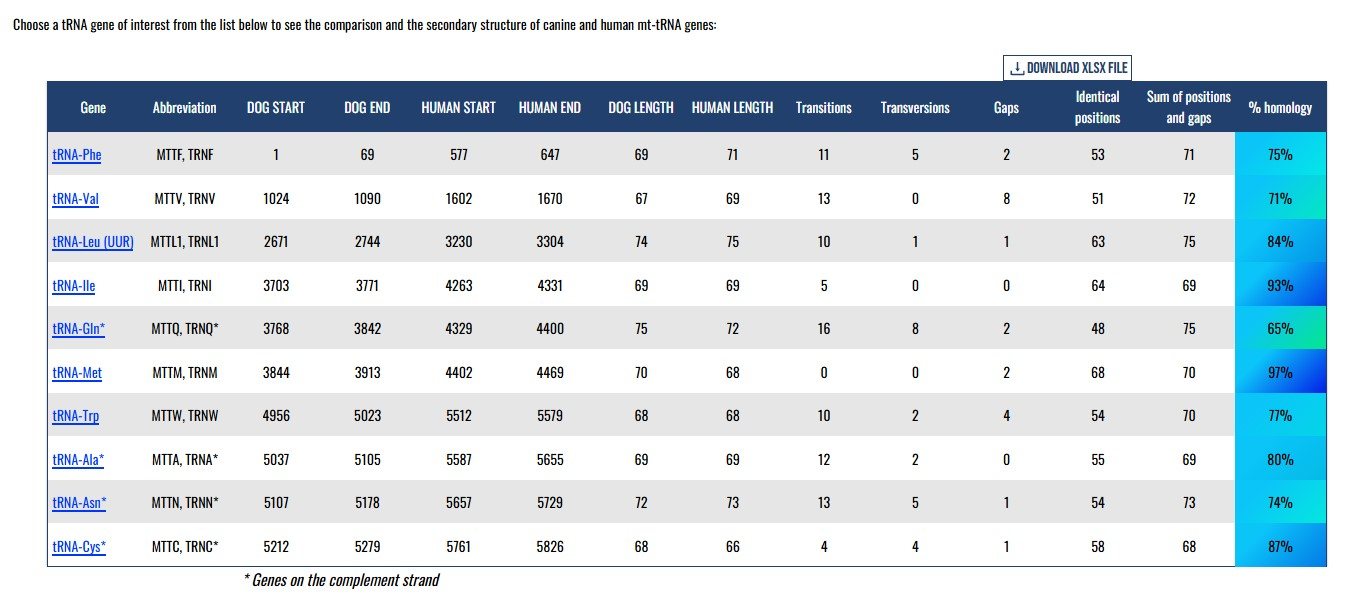


**(2b)**


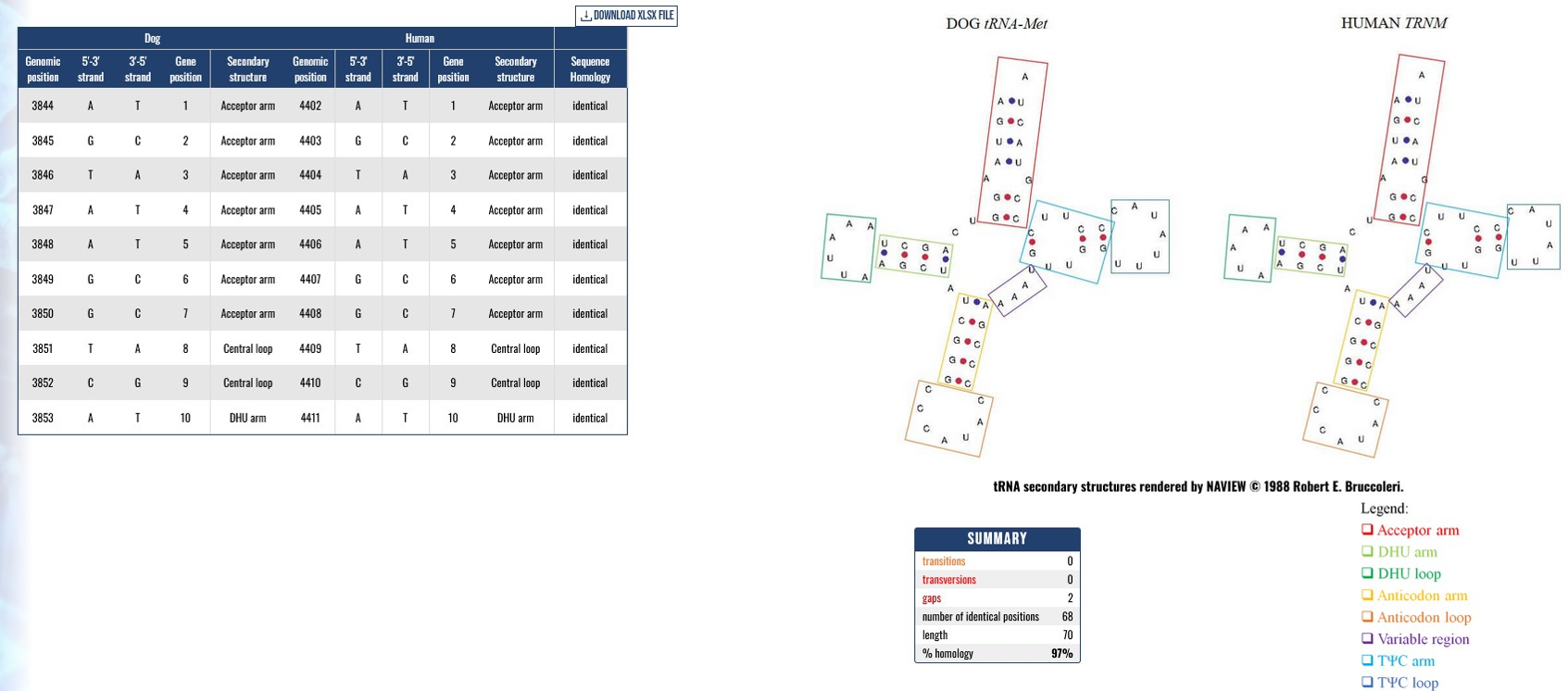


**Supplementary figures 2a**. tRNA properties website screen. **2b.** Example of a report for one of the tRNA-coding genes

**(3a)**


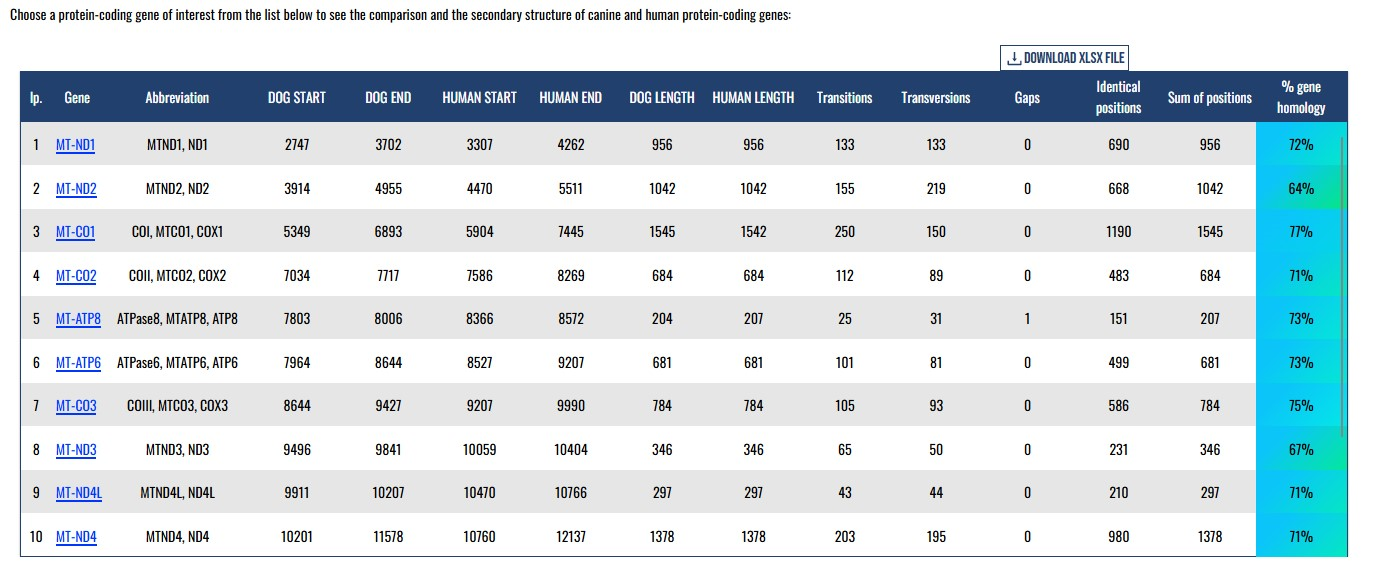


**(3b)**


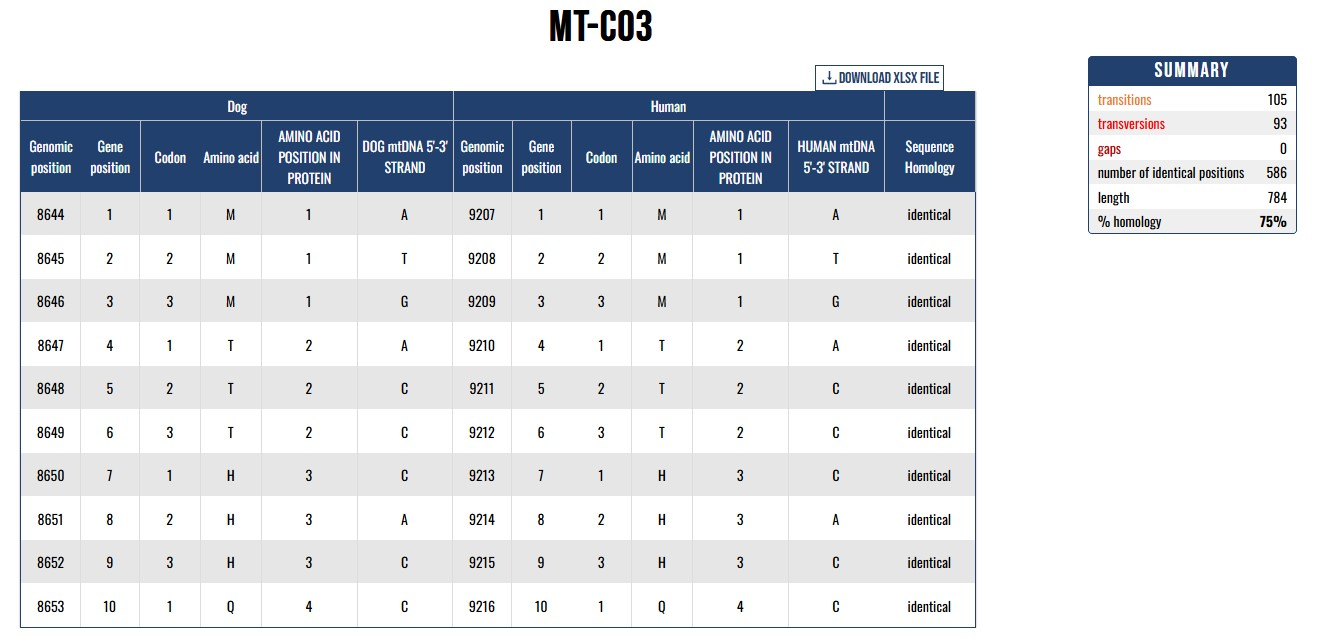


**Supplementary figures 3a.** Summary of mRNA properties for one of the protein-coding genes.

**3b.** Detailed comparison between human and dog mRNA sequences with the summary information

**(4a)**


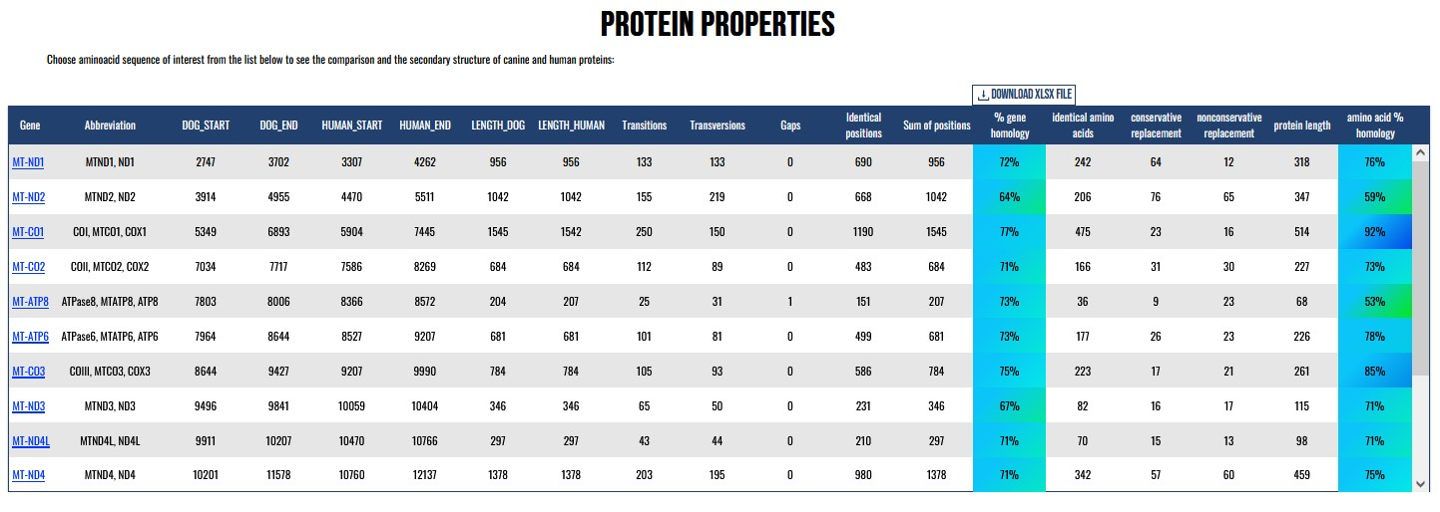


**(4b)**


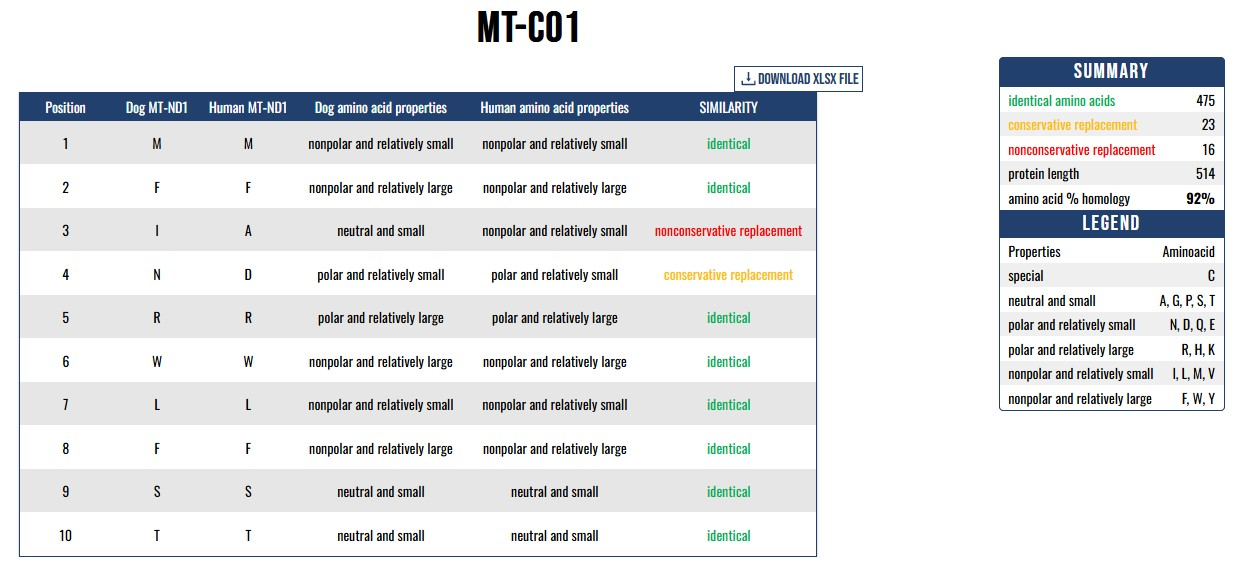


**Supplementary figures 4a.** Protein properties website screen. **4b.** Summary of protein properties for one of the proteins
