# Supplementary material for: Canis MitoSNP database: a functional tool useful for comparative analyses of human and canine mitochondrial genomes
Source: J Appl Genet. 2023 Jun 23;64(3):515–20. doi: 10.1007/s13353-023-00764-w (PMC10457218; doi:10.1007/s13353-023-00764-w)
Supplement: Supplementary file 2 — Supplementary file2 (DOCX 19 KB) [file 13353_2023_764_MOESM2_ESM.docx]

**Supplementary Table** Non-coding regions and their homological positions in the canine mitochondrial genome according to the sequence alignment analysis

| **Map locus** | **Description** | **Dog start** | **Dog end** | **Dog length** | **Human start** | **Human end** | **Human length** | **Transitions** | **Transversions** | **Gaps** | **Identical positions** | **Sum of positions** | **% homology** | **Explanation** |
| --- | --- | --- | --- | --- | --- | --- | --- | --- | --- | --- | --- | --- | --- | --- |
| **MT-3H** | mt3 H-strand control element | 16630 | 16637 | 8 | 384 | 391 | 8 | 0 | 1 | 0 | 7 | 8 | **88%** | based on the analysis of GenBank ref. seq. and sequence homology |
| **MT-3L** | L-strand control element | 15787 | 15794 | 8 | 16499 | 16506 | 8 | 0 | 0 | 0 | 8 | 8 | **100%** |  |
| **MT-4H** | mt4 H-strand control element | 15894 | 15902 | 9 | 371 | 379 | 9 | 2 | 0 | 0 | 7 | 9 | **67%** |  |
| **MT-5** | control element | 15565 | 15579 | 15 | 16194 | 16208 | 15 | 1 | 2 | 0 | 12 | 15 | **80%** |  |
| **MT-7SDNA** | 7S DNA |  |  |  | 16106 | 191 | 655 |  |  |  |  |  | N/D* | cannot be determined based on the alignment |
| **MT-ATT** | membrane attachment site |  |  |  | 15925 | 499 | 1144 |  |  |  |  |  |  |  |
| **MT-CR** | entire Control Region |  |  |  | 16024 | 576 | 1122 |  |  |  |  |  |  |  |
| **MT-CSB1** | Conserved sequence block 1 | 16098 | 16120 | 23 | 213 | 235 | 23 | 4 | 3 | 0 | 16 | 23 | **70%** | based on the analysis of GenBank ref. seq. and sequence homology |
| **MT-CSB2** | Conserved sequence block 2 | 16461 | 16477 | 17 | 299 | 315 | 17 | 1 | 1 | 0 | 15 | 17 | **88%** |  |
| **MT-CSB3** | Conserved sequence block 3 | 16518 | 16535 | 18 | 346 | 363 | 18 | 1 | 0 | 0 | 17 | 18 | **94%** |  |
| **MT-HPR** | replication primer |  |  |  | 317 | 321 | 5 |  |  |  |  |  | N/D* | cannot be determined based on the alignment |
| **MT-HSP1** | Major H-strand promoter |  |  |  | 545 | 567 | 23 |  |  |  |  |  |  |  |
| **MT-HSP2** | Minor H-strand promoter |  |  |  | 645 | 645 | 1 |  |  |  |  |  |  |  |
| **MT-Hum** | Humanin peptide | 2060 | 2132 | 73 | 2633 | 2705 | 73 | 7 | 3 | 0 | 63 | 73 | **86%** | based on the analysis of GenBank ref. seq. and sequence homology |
| **MT-HV1** | Hypervariable segment 1 |  |  |  | 16024 | 16383 | 360 |  |  |  |  |  | N/D* | cannot be determined based on the alignment |
| **MT-HV2** | Hypervariable segment 2 |  |  |  | 57 | 372 | 316 |  |  |  |  |  |  |  |
| **MT-HV3** | Hypervariable segment 3 |  |  |  | 438 | 574 | 137 |  |  |  |  |  |  |  |
| **MT-LSP** | L-strand promoter |  |  |  | 392 | 435 | 44 |  |  |  |  |  |  |  |

*Cont.*

| **MT-NC1** | non-coding nucleotides | 2745 | 2746 | 2 | 3305 | 3306 | 2 |  |  |  |  |  | N/A* | based on the analysis of GenBank ref. seq. |
| --- | --- | --- | --- | --- | --- | --- | --- | --- | --- | --- | --- | --- | --- | --- |
| **MT-NC2** |  | 3843 | 3843 | 1 | 4401 | 4401 | 1 |  |  |  |  |  |  |  |
| **MT-NC3** |  | 5024 | 5036 | 13 | 5580 | 5586 | 7 |  |  |  |  |  |  |  |
| **MT-NC4** |  | 5106 | 5106 | 1 | 5656 | 5656 | 1 |  |  |  |  |  |  |  |
| **MT-NC5** |  | 5348 | 5348 | 1 | 5892 | 5903 | 12 |  |  |  |  |  |  |  |
| **MT-NC6** |  | 6962 | 6965 | 4 | 7515 | 7517 | 3 |  |  |  |  |  |  |  |
| **MT-NC7** |  | 7718 | 7734 | 17 | 8270 | 8294 | 25 |  |  |  |  |  |  |  |
| **MT-NC8** |  | 7802 | 7802 | 1 | 8365 | 8365 | 1 |  |  |  |  |  |  |  |
| **MT-NC9** |  | 14179 | 14182 | 4 | 14743 | 14746 | 4 |  |  |  |  |  |  |  |
| **MT-NC10** | non-coding nucleotides |  |  |  | 15954 | 15955 | 2 |  |  |  |  |  | N/A* | not observed in the canine genome |
| **MT-OHR** | H-strand origin |  |  |  | 110 | 441 | 332 |  |  |  |  |  | N/D* | cannot be determined based on the alignment |
| **MT-OHR57** | H-strand origin |  |  |  | 57 | 57 | 1 |  |  |  |  |  |  |  |
| **MT-OLR** | L-strand origin | 5179 | 5215 | 37 | 5721 | 5798 | 78 | 7 | 3 | 3 | 68 | 81 | **84%** | positions from GenBank |
| **MT-RNR3** | 5S-like sequence | 2645 | 2669 | 25 | 3206 | 3229 | 24 | 4 | 4 | 2 | 16 | 26 | **62%** | based on the analysis of GenBank ref. seq. and sequence homology |
| **MT-TAS** | termination-associated sequence |  |  |  | 16157 | 16172 | 16 |  |  |  |  |  | N/D* | cannot be determined based on the alignment |
| **MT-TAS2** | extended termination-associated sequence | 15530 | 15587 | 58 | 16081 | 16138 | 58 | 6 | 8 | 6 | 42 | 62 | **68%** | based on the analysis of GenBank ref. seq. and sequence homology |
| **MT-TER** | Transcription terminator | 2669 | 2696 | 28 | 3229 | 3256 | 28 | 5 | 0 | 1 | 23 | 29 | **79%** |  |
| **MT-TFH** | mtTF1 binding sites |  |  |  | 523 | 550 | 28 |  |  |  |  |  | N/D* | cannot be determined based on the alignment |
| **MT-TFL** |  |  |  |  | 418 | 445 | 28 |  |  |  |  |  |  |  |
| **MT-TFX** |  |  |  |  | 233 | 260 | 28 |  |  |  |  |  |  |  |
| **MT-TFY** |  |  |  |  | 276 | 303 | 28 |  |  |  |  |  |  |  |

* N/D – Not Determined, N/A - Not Applicable
